# Transcriptomic and photosynthetic analyses of *Synechocystis* sp. PCC6803 and *Chlorogloeopsis fritschii* sp. PCC6912 exposed to an M-dwarf spectrum under an anoxic atmosphere

**DOI:** 10.3389/fpls.2023.1322052

**Published:** 2024-01-18

**Authors:** Mariano Battistuzzi, Maria Silvia Morlino, Lorenzo Cocola, Livio Trainotti, Laura Treu, Stefano Campanaro, Riccardo Claudi, Luca Poletto, Nicoletta La Rocca

**Affiliations:** ^1^ National Council of Research of Italy, Institute for Photonics and Nanotechnologies (CNR-IFN), Padua, Italy; ^2^ Department of Biology, University of Padua, Padua, Italy; ^3^ Center for Space Studies and Activities (CISAS), University of Padua, Padua, Italy; ^4^ National Institute for Astrophysics, Astronomical Observatory of Padua (INAF-OAPD), Padua, Italy; ^5^ Department of Mathematics and Physics, University Roma Tre, Rome, Italy

**Keywords:** cyanobacteria, laboratory simulation experiments, M-dwarf spectrum, anoxia, transcriptomic analysis, RNA-Seq, FaRLiP

## Abstract

**Introduction:**

Cyanobacteria appeared in the anoxic Archean Earth, evolving for the first time oxygenic photosynthesis and deeply changing the atmosphere by introducing oxygen. Starting possibly from UV-protected environments, characterized by low visible and far-red enriched light spectra, cyanobacteria spread everywhere on Earth thanks to their adaptation capabilities in light harvesting. In the last decade, few cyanobacteria species which can acclimate to far-red light through Far-Red Light Photoacclimation (FaRLiP) have been isolated. FaRLiP cyanobacteria were thus proposed as model organisms to study the origin of oxygenic photosynthesis as well as its possible functionality around stars with high far-red emission, the M-dwarfs. These stars are astrobiological targets, as their longevity could sustain life evolution and they demonstrated to host rocky terrestrial-like exoplanets within their Habitable Zone.

**Methods:**

We studied the acclimation responses of the FaRLiP strain *Chlorogloeopsis fritschii* sp. PCC6912 and the non-FaRLiP strain *Synechocystis* sp. PCC6803 to the combination of three simulated light spectra (M-dwarf, solar and far-red) and two atmospheric compositions (oxic, anoxic). We first checked their growth, O_2_ production and pigment composition, then we studied their transcriptional responses by RNA sequencing under each combination of light spectrum and atmosphere conditions.

**Results and discussion:**

PCC6803 did not show relevant differences in gene expression when comparing the responses to M-dwarf and solar-simulated lights, while far-red caused a variation in the transcriptional level of many genes. PCC6912 showed, on the contrary, different transcriptional responses to each light condition and activated the FaRLiP response under the M-dwarf simulated light. Surprisingly, the anoxic atmosphere did not impact the transcriptional profile of the 2 strains significantly. Results show that both cyanobacteria seem inherently prepared for anoxia and to harvest the photons emitted by a simulated M-dwarf star, whether they are only visible (PCC6803) or also far-red photons (PCC6912). They also show that visible photons in the simulated M-dwarf are sufficient to keep a similar metabolism with respect to solar-simulated light.

**Conclusion:**

Results prove the adaptability of the cyanobacterial metabolism and enhance the plausibility of finding oxygenic biospheres on exoplanets orbiting M-dwarf stars.

## Introduction

Cyanobacteria, a phylum of photoautotrophic prokaryotes, appeared during the Archean (~3.8 - 2.5 Ga) and were the first to evolve oxygenic photosynthesis (Van Kranendonk et al., 2012; Fournier et al., 2021; Robbins et al., 2023), greatly innovating life on Earth. At first cyanobacterial distribution was possibly local and confined to the subsurface (Havig and Hamilton, 2019), due to the intense radiation environment of the Archean (Ratner and Walker, 1972). In these habitats cyanobacteria experienced a light spectrum depleted of visible (400 - 700 nm) and enriched in far-red light (701 - 750 nm) (Gan and Bryant, 2015), and possibly among them some could have had the capability to utilize far-red light as suggested by Gisriel and coworkers (Gisriel et al., 2022).

Later, as O_2_ and O_3_ levels started to rise (Ligrone, 2019) protecting the Earth’s surface from UV radiations, they eventually spread on the whole planet, optimizing in the process their photosynthetic apparatus to harvest the more energetic visible wavelengths of the Sun’s spectrum (Oliver et al., 2023). In the early Proterozoic (~2.4 – 2.0 Ga), the accumulation of O_2_ produced by cyanobacteria led to the Great Oxidation Event (GOE), changing Earth’s atmosphere forever, causing the first mass extinction on our planet but also the subsequent evolution of modern living organisms. Since GOE, oxygenic photosynthetic organisms have become the primary producers on Earth (Raven, 2009), feeding on the light harvested by the Sun, and O_2_ has become a permanent component of the atmosphere (Lyons et al., 2014), even if its concentration has risen and fallen multiple times throughout eons until ~0.2 Ga when it stabilized to the current 21% (Catling and Zahnle, 2020). Up to recent years, oxygenic photosynthetic organisms were believed to utilize only the visible light abundant on the surface of the planet. However, many habitats on Earth, characterized by low visible light availability, still host oxygenic photosynthetic microorganisms. In stromatolites, endolithic environments, sediments or in caves, as well as in microbial mats or in coastal water columns, most visible light is physically scattered or absorbed by the top layers of phototrophs through chlorophyll *a*, while far-red-enriched light gets to the lower layers (Kasperbauer, 1987; Gilbert et al., 2001; Chen et al., 2012; Ramírez-Reinat and Garcia-Pichel, 2012; Behrendt et al., 2015; Gan and Bryant, 2015). In these environments, spectrally similar to those that probably allowed oxygenic photosynthesis evolution, some species of cyanobacteria were found to harvest far-red photons by acclimating through the so-called Far-Red Light Photoacclimation (FaRLiP) (Gan et al., 2014b). FaRLiP cyanobacteria under far-red light synthesize chlorophylls *d* and *f* (Chl *d*, Chl *f*) and far-red forms of allophycocyanin (AP). They substitute core protein subunits of the photosystem I (PSI), photosystem II (PSII) and the phycobilisome (PBS) with far-red paralogs, and modify the morphology of the PBS antennae to absorb the far-red light (Gan et al., 2014b; Gan et al., 2014b; Zhao et al., 2015; Li et al., 2016; Ho et al., 2017a; Ho et al., 2017b). The FaRLiP response was first described in the strain *Leptolyngbya* sp. JSC-1 (Gan et al., 2014b) but was in a short time experimentally identified in several species (Gan et al., 2014b) and is believed to be widespread globally (Zhang et al., 2019; Antonaru et al., 2020; Kühl et al., 2020). The FaRLiP response is controlled by the homonymous conserved gene cluster, induced through a set of regulatory genes (*rfpABC*) encoded within it (Zhao et al., 2015). The FaRLiP cluster encodes generally 19-20 genes, even if at least one functional cluster containing only 15 genes was discovered (Billi et al., 2022). A typical FaRLiP gene cluster encodes the Chl *f* synthase (ChlF) and the FR-paralogs of PSI, PSII, and PBS (Gan et al., 2014b; Gan et al., 2014b; Zhao et al., 2015; Ho et al., 2016; Ho et al., 2017a; Ho et al., 2017b). The cluster probably encodes Chl *d* synthase, but to date, the responsible gene remains unidentified (Yoneda et al., 2016; Ho and Bryant, 2019). The discovery of FaRLiP cyanobacteria matches the interests of the astrobiology community. Given the fundamental role of oxygenic photosynthesis on Earth, astrobiologists have wondered if this process could function and drive primary productivity around other stellar types. Prime targets for this research have become M-dwarfs, long-lived stars (Adams et al., 2004), abundant in our galaxy (70 to 75% of the stars in the Milky Way) (Henry et al., 1994), and which demonstrated to host terrestrial-like exoplanets (Bonfils et al., 2013; Kopparapu et al., 2013; Hsu et al., 2020) within their Habitable Zone (Kasting et al., 1993). These stars are cooler and fainter than the Sun (Pecaut and Mamajek, 2013), consequently, they present spectra with high emission in the far-red but very low emission in the visible, providing a spectral environment surprisingly similar to those where FaRLiP species live. Recently, we verified experimentally the ability of cyanobacteria capable or incapable of FaRLiP to survive, grow and have a photosynthetic activity under M-dwarfs simulated light conditions (Claudi et al., 2021); moreover, we discovered that the FaRLiP cyanobacterium *Chlorogloeopsis fritschii* sp. PCC6912 (hereafter, PCC6912) was able to acclimate through FaRLiP while growing under those light conditions (Battistuzzi et al., 2023). After testing the physiological responses of the strains to a simulated M-dwarf light condition under a terrestrial atmosphere, we combined the irradiation treatment with oxic (75% N_2_, 20% O_2_, 5% CO_2_) and anoxic (95% N_2_ and 5% CO_2_) atmospheres. The composition of the anoxic atmosphere was chosen as a plausible condition of an Archean Earth where life had already developed, cyanobacteria had just evolved and CO_2_ levels were in the order of 5% by volume, while no O_2_ was yet present in the atmosphere (Catling and Zahnle, 2020; Kaltenegger et al., 2020). In these conditions, we studied through a biochemical and transcriptomic approach the responses of the strains.

## Materials and methods

### Experimental design

In this study two different cyanobacterial strains were utilized, respectively able (PCC6912) and unable (*Synechocystis* sp. PCC6803, hereafter, PCC6803) of FaRLiP response. Acclimation experiments to the M-dwarf irradiation combined with oxic and anoxic atmospheres were performed and lasted 48 hours. The timing of 48 hours was selected based on the work by Ho and Bryant (2019), in which they reported the maximum activation of the FaRLiP response at the transcriptional level after 48 hours of exposure to far-red light. For each combination of organisms (2), light regimes (3) and atmosphere compositions (2) (**Supplementary Table 1**), 3 independent biological replicates were obtained (N = 3). Acclimation experiments were performed inside the experimental setup which is extensively described in Battistuzzi et al. (2020); Battistuzzi et al. (2023). Briefly, the setup is composed of 3 cabinets, each including a flatbed kept at constant temperature and illuminated by a different light source. On each flatbed, a growth chamber is positioned (Atmosphere Simulator Chamber, or ASC), which is utilized to combine light irradiation and exposure to oxic and anoxic atmospheres. The ASC is kept at constant temperature and pressure and has CO_2_ and O_2_ sensors to monitor in real-time the gas exchanges of cyanobacteria with the atmosphere remotely. For the acclimation experiments, the light spectrum of each light source was set at 30 µmol m^-2^ s^-1^ in the range between 380 and 780 nm and checked through a spectrometer (LI-COR 180, LI-COR). The light spectra utilized were a simulated M-dwarf light spectrum (termed “M7”), a simulated solar light spectrum (termed “SOL”), and a 730 nm far-red light (termed “FR”) (Battistuzzi et al., 2023). Two initial atmospheric compositions were utilized in this study: the first is an oxic, terrestrial-like atmosphere enriched in carbon dioxide termed “ATM TER” (composed of 75% N_2_, 20% O_2_, 5% CO_2_), the second is an anoxic atmosphere termed “ATM MOD” (composed of 95% N_2_ and 5% CO_2_). Before the start and at the end of each experiment, optical density, dry weight and pigment content measurements were made to investigate the organisms’ growth. Moreover, by utilizing the O_2_ sensors of the ASC, the O_2_ production of the strains directly exposed under the three light sources was measured. Finally, to characterize the initial phases of the FaRLiP process in the FaRLiP strain, *in vivo* absorption spectroscopy and chl *d* and *f* detection through HPLC analyses were carried out. The entire experimental campaign was conducted between June 2020 and June 2021.

### Cultivation conditions

The selected strains, PCC6803 and PCC6912, were acquired from the Pasteur Culture Collection (PCC, France) in April 2019. They were maintained in liquid cultures in a climatic chamber at 30 ± 0.5°C, exposed to a terrestrial atmosphere and a continuous cool white, fluorescent light of 30 μmols m^−2^ s^−1^ (L36W-840, OSRAM). Both strains were maintained in a BG-11 liquid medium (Rippka et al., 1979). Cryo-storage of the strains was not employed.

Before each experiment, cells from maintenance cultures were pre-inoculated in flasks at an optical density at 750 nm (OD_750_) of 0.2 in a final volume of 100 mL and were exposed to the SOL light spectrum at an intensity of 30 μmols m^−2^ s^−1^ (380 – 780 nm) until they reached exponential phase: OD_750_ of about 0.5 for PCC6803 and OD_750_ of about 0.9 for PCC6912. The cultures of the pre-inoculum were centrifuged at 3500 g for 5 min and resuspended to adjust the OD_750_ to 0.6 with fresh BG-11 medium. For each combination of light condition, organism and atmospheric condition (**Supplementary Table 1**), 70 mL of these cultures were directly poured into a previously sterilized glass Petri dish and put inside the ASC for 48 hours. Each experiment listed in **Supplementary Table 1** was performed in biological triplicate (N = 3).

### Dry weight determination

To measure the dried biomass concentration, 10 mL of culture were diluted with 20 mL of deionized water and filtered with a vacuum flask on 0.45 µm nitrocellulose filters (Sigma), previously dried in a heater at 70°C for at least 3 h and weighed. Filters with cyanobacteria were put again in the heater at 70°C to dry and weighed at least after 24 h. Dry weight (DW) was calculated as follows:


$$Dry\;Weight\;\left[\frac{g}{L}\right]=\;\frac{\left(Filter\;with\;cyanobacteria\;\left[g\right]-Empty\;filter\left[g\right]\right)}{Volume\;of\;culture\;filtered\;\left[mL\right]}\times 1000$$

### *In vivo* absorption analysis

For *in vivo* absorption measurements, 1.5 mL of culture were centrifuged twice at 1400 g for 5 min (Sigma Centrifuge 3K15). The supernatant was discarded, and the pellet was homogenized with a pestle and then resuspended in 600 µL of fresh BG-11 medium. The suspension was then analyzed through a spectrophotometer (Agilent Cary 300 UV-VIS), using optical glass cuvettes and exposing to the ray their opaque side to correct scattering (Gan et al., 2014b).

### Pigment content determination

To evaluate the chlorophyll *a* and total carotenoid content, 2 mL of culture were centrifuged for 10 min at 17500 g. The supernatant was discarded, and the pellet was solubilized in 1 mL of DMF (N,N′-dimethylformamide). Samples were kept at 4°C in the dark for at least 24 h, to allow the extraction of lipophilic pigments. Pigment spectra were recorded with a spectrophotometer (Agilent Cary 300 UV-Vis) by using optical glass cuvettes. The concentrations of chlorophyll *a* and total carotenoids were calculated using the Moran equations for DMF (Moran, 1982).

### HPLC analysis

For liquid chromatographic analyses, 4 mL of culture were centrifuged twice at 17500 g, for 5 min, at 4°C. The supernatant was discarded and to the pellet were added glass beads (150-212 µm, Sigma) and 20 µL of acetone 90%. Samples were disrupted through a Bead-Beater (Biospec Products) with three cycles of rupture for 10 s at 3500 OPM (oscillations per minute) followed by 30 s in ice. 180 µL of acetone 90% were then added to the mixture and another cycle of Bead-Beater was repeated for 4 s. The samples were centrifuged twice at 20.000 g, for 5 min, at 4°C, and the supernatants were kept aside. To ensure complete extraction of the pigments, a second extraction was performed by adding 800 µL of acetone 90% to the pellets, by vortexing and by centrifuging the mixture as before. The obtained extracts were added to the previous extraction to obtain about 1 mL of final pigment extract and were stored at -20°C until analysis. For the analyses, an Agilent 1100 series LC system was utilized. The stationary phase was a column (length 250 mm, diameter 4 mm) internally filled with 5 µm silica particles coated by C-18 atom chains (Merck Lichrospher 100 RP). The mobile phase was composed of two solutions: A) methanol/acetonitrile/HPLC-grade H_2_O, in the ratio 42:33:25; B) methanol/acetonitrile/ethyl acetate, in the ratio 50:20:30. Solutions A and B were eluted in the column following the protocol reported in Gan et al. (2014b), optimized for the detection of Chl *d* and *f*. During each run, the detector registered the absorption levels of each pigment eluted from the column when illuminated by a 705 nm source light.

### Gas exchange measurements

The cyanobacteria photosynthetic rate was measured along the 48 h when the organisms were directly exposed to the respective growth light conditions inside the ASC. Raw data of O_2_ levels registered in the chamber were elaborated through Matlab (MathWorks) to obtain the O_2_ production expressed in micromoles by applying the ideal gas law (Battistuzzi et al., 2020).

### Statistical analysis on growth, O_2_ production and pigment content

Statistical analyses were performed on Graph Pad Prism v7.0 (GraphPad software). Data were calculated as the mean ± standard deviation of 3 biological replicates. One-way ANOVA technique (assuming the Gaussian distribution of data) followed by Tukey’s multiple comparison test (significance was set at p< 0.05) was used to determine the significance of 1) comparisons between different light spectra (M7, SOL, FR) when considering the same atmosphere and strain; 2) comparisons between different atmosphere compositions (ATM TER, ATM MOD) when considering the same light spectrum and strain.

### DNA extraction

For the DNA extraction of PCC6912, 2 ml of culture from a maintenance line in the exponential phase of growth were harvested and pelleted at 21400 g for 5 min. The supernatant was discarded and to the pellet were added 200 µl of cold buffer TEN (100 mM Tris-HCl pH 8, 50 mM EDTA, 500 mM NaCl) and an equal volume of glass beads. Samples were disrupted through a Bead-Beater with four cycles of rupture for 30 s at 3500 OPM followed by 1 min in ice. After the addition of 35 µl of SDS 20%, the mixture was incubated in a thermoblock for 5 min at 65°C. 130 µl of KOAc 5M were then added, and the samples were put in ice for 5 min. After centrifugation at 21400 g for 10 min, the supernatant was transferred to a new test tube containing 500 µl of isopropanol 100%, mixed and incubated for 10 min at -20°C. After this, the pellet was washed twice with decreasing volumes of EtOH 70%. Afterwards, the pellet was briefly left to dry under an air flow and then resuspended in 50 µl of water. The extracted DNA was kept at -20°C before analyses.

### RNA extraction, isolation and purification

For the RNA extraction, depending on the cell concentration and strain, 10 to 15 mL of culture were harvested and centrifuged at 1500 g for 10 min at 4°C. The supernatant was discarded, and cell pellets were rapidly frozen in nitrogen liquid and kept at -80°C until further analyses. The whole process was performed in roughly 30 min and under a dim green light. For total RNA isolation, 50 µl of TRI Reagent (Sigma) and an equal volume of glass beads were added to the pellets. Cells were broken through the Bead-Beater with three cycles of rupture for 30 s at 3500 OPM followed by 1 min in ice. After the addition of 950 µl of TRI Reagent, a last cycle of rupture through the Bead-Beater was performed. The samples were then vortexed for 15 s and left 5 min at room temperature. 200 µl of chloroform were added to the mixture and the samples were mixed vigorously for 15 s and then kept 10 min at room temperature. The samples were then centrifuged at 12000 g for 15 min at 4°C. The aqueous phase containing the total RNA was transferred to a new test tube containing 500 µl of Isopropanol 100%, mixed and left 10 min at room temperature. The samples were centrifuged at 12000 g for 10 min at 4°C to precipitate the RNA as a pellet. The pellet was then washed once with 1 ml of EtOH 75%. After vortexing and centrifugation at 12000 g for 15 min at 4°C, the samples were left to dry for 5-10 min, then they were resuspended in 50 µl of water RNase-free (Sigma). A first quantification of the raw total RNA extracts was performed spectrophotometrically with a Nanodrop 2000/2000c (ThermoFisher Scientific). For the RNA purification, the RNA Clean & Concentrator™-5 (Zymo Research) was used. DNase I (ThermoFisher Scientific) treatment of the samples and RNA purification were performed following the manufacturer’s protocol. After purification, samples were quantified again through Nanodrop and their quality was assessed with an RNA Bioanalyzer (Agilent), obtaining RNA Integrity Numbers (RIN) > 8.

### Nucleic acid processing and sequencing

Extracted DNA was processed to prepare libraries using the Nextera DNA Flex Library Prep Kit (Illumina Inc.). Sequencing was performed on the Illumina Novaseq 6000 platform (2 × 150, paired-end, Illumina Inc.) at the NGS facility of the Biology Department (University of Padova, Italy). On average, the sequencing run yielded 6.4 million read pairs per sample. To prepare DNA for Nanopore sequencing, an enrichment in large fragments was performed with AMPure beads (Beckman Coulter) at the concentration of 0.7x. The purified DNA was quantified with a Qubit fluorometer (ThermoFisher Scientific) and used to prepare a rapid barcoding library (SQK-RBK004, Oxford Nanopore Technology), according to the manufacturer’s instructions. The genomes were sequenced using a MinION platform, with an R10 flow cell (FLO-MIN106D, Oxford Nanopore Technology). Raw FAST5 files were base-called and barcode-split with Guppy (v. 5.0.11), yielding reads in FASTQ format.

For RNA sequencing, samples were treated with the QIAseq FastSelect kit (Qiagen) to mask ribosomal RNAs. Subsequently, libraries were prepared with the Illumina Stranded mRNA Prep (Illumina Inc.) and sequencing was carried out on the Illumina Novaseq 6000, similarly to what was described for DNA Illumina sequencing.

### Bioinformatic analyses

Genomic Nanopore sequences of PCC6912 were assembled using Canu (v. 2.2) (Koren et al., 2017). The estimated genome size was set to 7.7 Mbp. The assembly was polished using Illumina reads, which were preprocessed with Trimmomatic (Bolger et al., 2014) v0.39 using options “-phred33 LEADING:20 TRAILING:20 SLIDINGWINDOW:4:20 MINLEN:70” and clipped with BBDuk v38.86 to remove adapters. Subsequently, Illumina reads were aligned to the assembly using Bowtie2 (v. 2.4.4) (Langmead and Salzberg, 2012) and, subsequently, performing correction with Pilon (v. 1.24) (Walker et al., 2014). Assembly quality metrics were calculated with Quast (v. 5.0.2) (Gurevich et al., 2013). The assembled and polished genome was compared to the reference genome available in NCBI (GenBank ID: GCA_003990575.1, Will et al., 2019) using Mauve (Darling et al., 2004). The *de novo* assembly was aligned to its reference with progressiveMauve and assembly contigs were re-ordered to match the reference. Furthermore, average nucleotide identity (ANI) was calculated with dRep (v. 3.2.2) (Olm et al., 2017) in order to ascertain strain correspondence. Genome quality in terms of completeness and contamination was assessed with CheckM2 v0.1.2 (Chklovski et al., 2023) using default parameters (**Supplementary Table 3**). The genome sequence for PCC6912 used in this work has been deposited at DDBJ/ENA/GenBank under the accession JAWJEG000000000. The version described in this paper is version JAWJEG010000000. The GFF annotation file of the reference genome of PCC6803 was downloaded from NCBI and used for subsequent analyses (GenBank ID: GCA_000009725.1, Kaneko et al., 2003). Annotations from the reference PCC6912 genome were downloaded from NCBI in GFF format and transferred to the *de novo* assembly using Liftoff (v. 1.6.1) (Shumate and Salzberg, 2021). Unique regions not present in the reference genome were identified with AGEnt (v. 0.3.1), setting the minimum percent identity to 99 and the minimum length of unique regions to 500. A *de novo* gene prediction was carried out on these regions with Prodigal (v. 2.6.3) (Hyatt et al., 2010), and the predicted genes were functionally annotated with eggNOG (v. 2.0.1) (Cantalapiedra et al., 2021). Newly predicted genes were checked and discarded if redundant with other transferred features. The presence of genes of interest, such as the FaRLiP and LoLiP clusters, was checked with BLAST (v. 2.12.0+) (Altschul et al., 1990) and the coordinates of BLAST hits were ensured to match existing annotations in the GFF file. RNA-seq Illumina paired-end reads were aligned to the genomes with Bowtie2. Read counts for each gene were obtained from the alignment files and GFF annotations by using BEDTools. Read counts were imported in R (v. 4.1.2) and analyzed with DESeq2 (v. 3.14) (Love et al., 2014), setting the log fold change (LFC) threshold at 1 and q-value threshold at 0.05 after Benjamini-Hochberg correction. The following comparisons were made: (i) same light, different atmosphere; (ii) same atmosphere, different light. R library ggplot2 was used to generate PCA plots from normalized gene expression values.

## Results and discussion

### Effect of different light and atmosphere conditions on cyanobacterial growth and O_2_ accumulation

**Figure 1:**
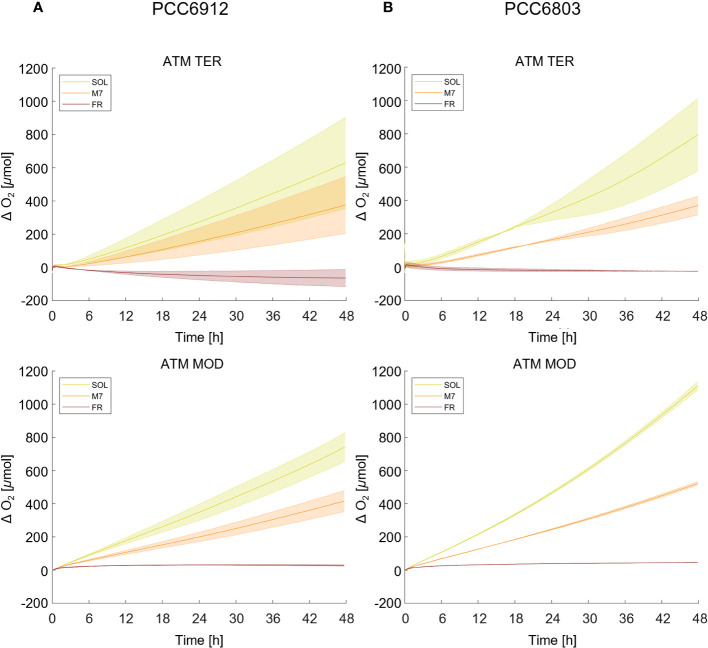
O_2_ evolution of PCC6912 **(A)** and PCC6803 **(B)** in ATM TER and ATM MOD over 48 h of experiment. T_0_ was set at 3.600 s to exclude the initial gas equilibration period inside the chamber. Bold lines represent the average of different biological replicates, with standard deviations reported as transparent areas. SOL, Solar light; M7, M-dwarf light; FR, Far-red light; ATM TER, oxic atmosphere; ATM MOD, anoxic atmosphere.

Growth of the cyanobacterial strains under the combined light and atmosphere conditions was first monitored through the rise of O_2_ gas levels inside the chamber (**Figure 1**; **Table 1**). In both strains, regardless of the atmosphere, SOL O_2_ production was the highest after 48 hours, followed by M7. FR never led to a relevant O_2_ production and instead, a consumption of O_2_ was observed in the ATM TER condition. PCC6912, within each light condition, evolved similar amounts of O_2_ in ATM TER and ATM MOD conditions. Instead, PCC6803 under SOL condition evolved significantly more O_2_ in ATM MOD than in ATM TER conditions (**Table 1**). Dry biomass and pigment content measurements were mostly concordant with the trends of O_2_ accumulation (**Supplementary Figure 1**; **Supplementary Table 2**). As expected from previous reports (Battistuzzi et al., 2023), PCC6803 showed an overall faster growth than PCC6912. Indeed, physiological data show that PCC6912 grew similarly in M7 and SOL, despite the differences in O_2_ evolution. PCC6803 showed a similar growth regardless of the atmospheric compositions tested, with SOL resulting in the highest growth, followed by M7. Both strains showed no detectable growth in FR. These results are clear also by visual inspection of the cultures before and after acclimation (**Supplementary Figure 2**).

**Table 1:** O_2_ production of PCC6912 and PCC6803 strains at 48 h in all conditions tested.

	O_2_ production at 48 h [µmol of O_2_]					
	ATM TER			ATM MOD		
	SOL	M7	FR	SOL	M7	FR
**PCC6912**	645.7 ± 195.9^c,d^	370.9 ± 121.4^c,e^	-68.7 ± 37.5^f^	826.8 ± 158.4^d^	449.3 ± 62.8^e^	29.9 ± 4.3^f^
**PCC6803**	808.0 ± 154.3	378.9 ± 41.6^a^	-26.7 ± 4.8^b^	1071.7 ± 72.1	522.3 ± 8.2^a^	44.2 ± 3.7^b^

The same letters highlight no significant differences between light conditions and atmospheric conditions within the same strain. (one-way ANOVA, p-value< 0,05). SOL, Solar light; M7, M-dwarf light; FR, Far-red light; ATM TER, oxic atmosphere; ATM MOD, anoxic atmosphere.

### Biochemical and spectroscopical assessment of FaRLiP response in *C. fritschii* PCC6912

**Figure 2:**
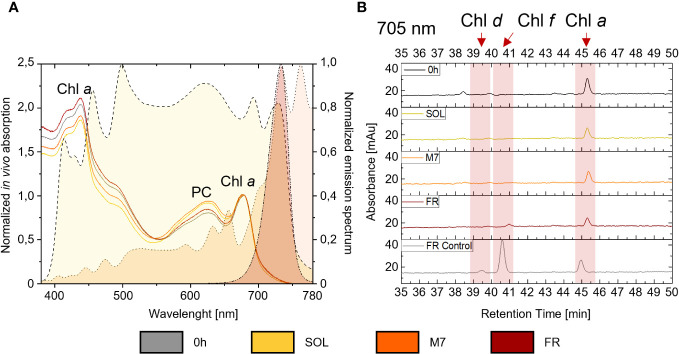
**(A)** on the right y-axis are plotted the normalized emission spectra of the light simulators utilized in the study. A dashed line with yellow fill pattern, a dotted line with orange fill pattern, and a dash-dot-dot line with dark red fill pattern are used respectively for the solar, M-dwarf, and far-red light spectra; on the left y-axis is plotted an example of *in vivo* absorption spectra of PCC6912 grown in the three light conditions under ATM TER; *in vivo* absorption spectra are normalized at 680 nm, while emission spectra of the light simulators are normalized to their respective peak emission in the range 380-780 nm; **(B)** example HPLC chromatograms at 705 nm of PCC6912 after 48 h of exposure to the different light conditions under ATM TER. Retention times for chlorophylls *a*, *d*, and *f* are highlighted with a red band. In gray is reported a control sample acclimated to FR light for 4 days at the same light intensity tested in these experiments. SOL, Solar light; M7, M-dwarf light; FR, Far-red light; ATM TER, oxic atmosphere.

The synthesis of chlorophylls *d*, *f*, necessary to the activation of FaRLiP in PCC6912, and their detectability *in vivo*, were investigated both spectroscopically, through the *in vivo* absorption of the far-red absorbing pigments, and biochemically, by evaluating the synthesis of the pigments through HPLC analyses. Comparable results were obtained for both atmospheres (**Figure 2**; **Supplementary Figure 3**): after 48 hours, *in vivo* spectroscopic analyses did not reveal any absorption above 700 nm in M7 or FR due to Chl *d* or *f* synthesis. An increased absorption in the waveband 560-650 nm (phycobiliproteins) was however observed in M7 and SOL. HPLC analyses gave similar results: as expected, Chl *d* and *f* synthesis was not detected in SOL, but no synthesis of these pigments was detected after 48 hours in M7 either, even if this light has a far-red component. In FR, only a small amount of Chl *f* was detected after 48 hours and Chl *d* was below detection limits, while the same culture acclimated to FR for 4 days at the same light intensity tested in these experiments, used as reference of a normal FaRLiP response activation, showed higher synthesis of far-red harvesting pigments. The lack of growth in FR for both strains and their consequent lack of O_2_ production was expected. PCC6803 is incapable of utilizing FR, therefore no growth was observed in this light condition. PCC6912 instead, albeit able to harvest this light through FaRLiP, takes at least 3 days to exhibit a detectable response (Battistuzzi et al., 2023), therefore after only 48 hours the strain cannot support the harvesting of far-red photons since it has not reorganized the photosynthetic apparatus yet and has barely detectable levels of chlorophyll *f* (**Figure 2**).

### Transcriptional response to different light and atmospheric conditions

**Figure 3:**
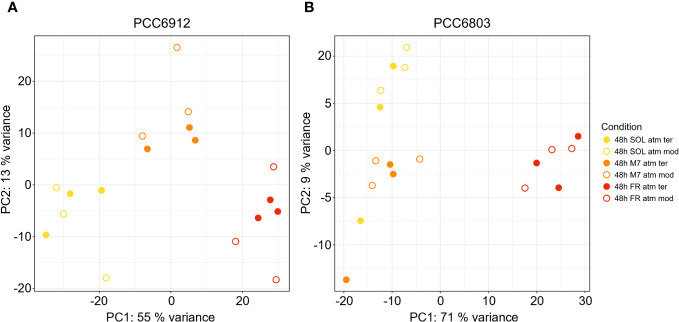
PCA plot generated from the expression profiles of PCC6912 **(A)** and PCC6803 **(B)**. Orange, red and yellow dots represent respectively M7, FR, SOL samples. Full and empty dots distinguish respectively ATM TER and ATM MOD samples. SOL, Solar light; M7, M-dwarf light; FR, Far-red light; ATM TER, oxic atmosphere; ATM MOD, anoxic atmosphere.

**Figure 4:**
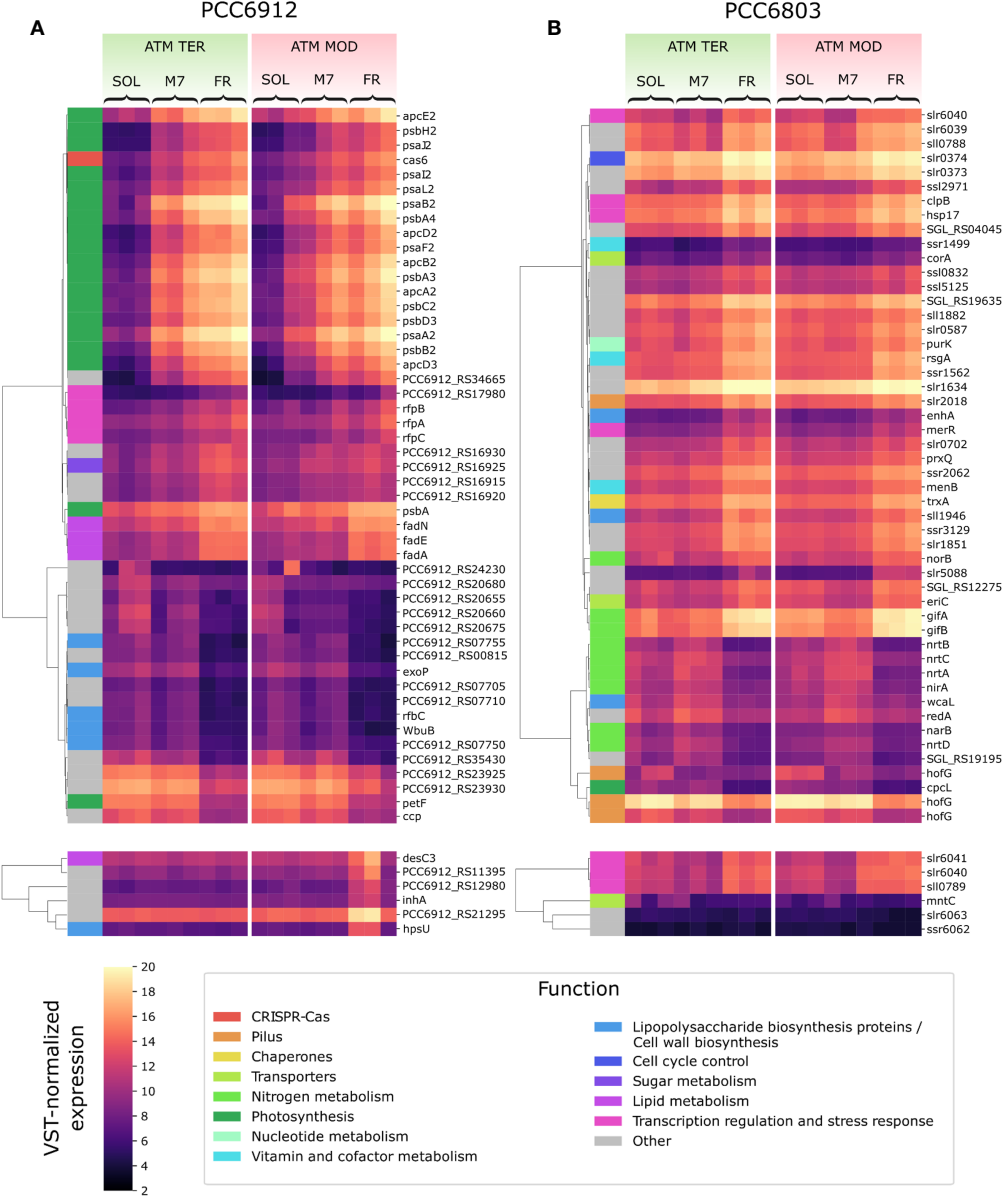
VST-normalized expression level of most variable genes in PCC6912 **(A)** and PCC6803 **(B)**. Genes are clustered by Pearson correlation. For each strain, the top panels show the 50 genes which showed the most variation when comparing light conditions, while the bottom panels show the 6 genes with the most variation across atmosphere conditions. Functional categories according to the KEGG database are reported for each gene. SOL, Solar light; M7, M-dwarf light; FR, Far-red light; ATM TER, oxic atmosphere; ATM MOD, anoxic atmosphere.

In order to confirm the FaRLiP activation in FR and possibly in M7 conditions for PCC6912, as well as the response of PCC6803 which is not endowed with FaRLiP, a transcriptomic analysis was carried out on both strains under all the combined atmospheric and light conditions. The analysis involved a total of 6163 genes for PCC6912 and 3738 genes for PCC6803 (**Supplementary files S1, S2, S3**). Before the transcriptomic analysis, both strains were re-sequenced. The quality of the new assembly obtained for PCC6912 was a remarkable improvement compared to assemblies currently available in NCBI for this strain (**Supplementary Figure 4**; **Supplementary Tables 3**, **4**), thus it was used for the subsequent analyses. On the contrary, for PCC6803 the NCBI reference genome was used. As described previously, biochemical and spectrophotometric analyses did not evidence relevant changes in the *in vivo* absorption properties of PCC6912 or in the synthesis of far-red-absorbing chlorophyll pigments after 48 hours of exposure to different lights and atmospheres. However, transcriptional analysis demonstrated that within this time frame, there were significant changes in the expression of several genes. PCC6912 shows diverse transcriptional profiles under the three light conditions. Exposure to FR triggers the strongest transcriptional responses, as expected, but significant differences in gene expression were detected also when comparing SOL and M7 (**Table 2**). A different scenario is observed in PCC6803, where M7 is not associated with significant changes in the transcriptome with respect to SOL, while FR samples present larger variations. This can be explained due to the presence of visible light within the M7 spectrum, which is enough for PCC6803 to photosynthesize and grow, as already demonstrated (Claudi et al., 2021; Battistuzzi et al., 2023). FR triggers more drastic responses since PCC6803 cannot utilize this light for photosynthesis. Finally, the effect of different atmospheres was not linked to major changes overall in the transcriptome, although some genes were differentially regulated (Q< 0.05) in PCC6912 under anoxic atmosphere in combination with FR light (**Table 2**). The principal component analysis (PCA) performed on the samples confirms these observations: PCC6912 samples are clustered according to light conditions, and the three clusters are spread along the first principal component; on the other hand, in PCC6803, SOL and M7 samples form a single cluster, while FR samples are neatly separated. Furthermore, in all strains and conditions, no clustering of the samples is observed according to atmospheric conditions (**Figure 3**). A detailed analysis of the genes and pathways which were affected by light and atmosphere conditions allowed to better characterize the responses of the two strains (**Figure 4**). Consistently with the PCA results, no major differences were observed in association with different atmospheric conditions for most genes. It seems that anoxia does not play a major role in the acclimation of both strains. The major differences were instead found when comparing different light spectra.

**Table 2:** Number of significantly up- and downregulated genes in comparisons between different light conditions under the same atmosphere.

Strain	PCC6912		PCC6803	
Regulation	Upregulated	Downregulated	Upregulated	Downregulated
**M7 vs SOL ATM TER**	19	0	0	0
**FR vs SOL ATM TER**	44	37	70	28
**FR vs M7 ATM TER**	6	14	62	30
**M7 vs SOL ATM MOD**	24	1	0	0
**FR vs SOL ATM MOD**	59	51	35	34
**FR vs M7 ATM MOD**	15	37	26	31
**ATM TER vs ATM MOD SOL**	0	0	0	0
**ATM TER vs ATM MOD FR**	0	6	0	0
**ATM TER vs ATM MOD M7**	0	0	0	0

SOL, Solar light; M7, M-dwarf light; FR, Far-red light; ATM TER, oxic atmosphere; ATM MOD, anoxic atmosphere.

### Most differentially regulated genes in PCC6912

**Figure 5:**
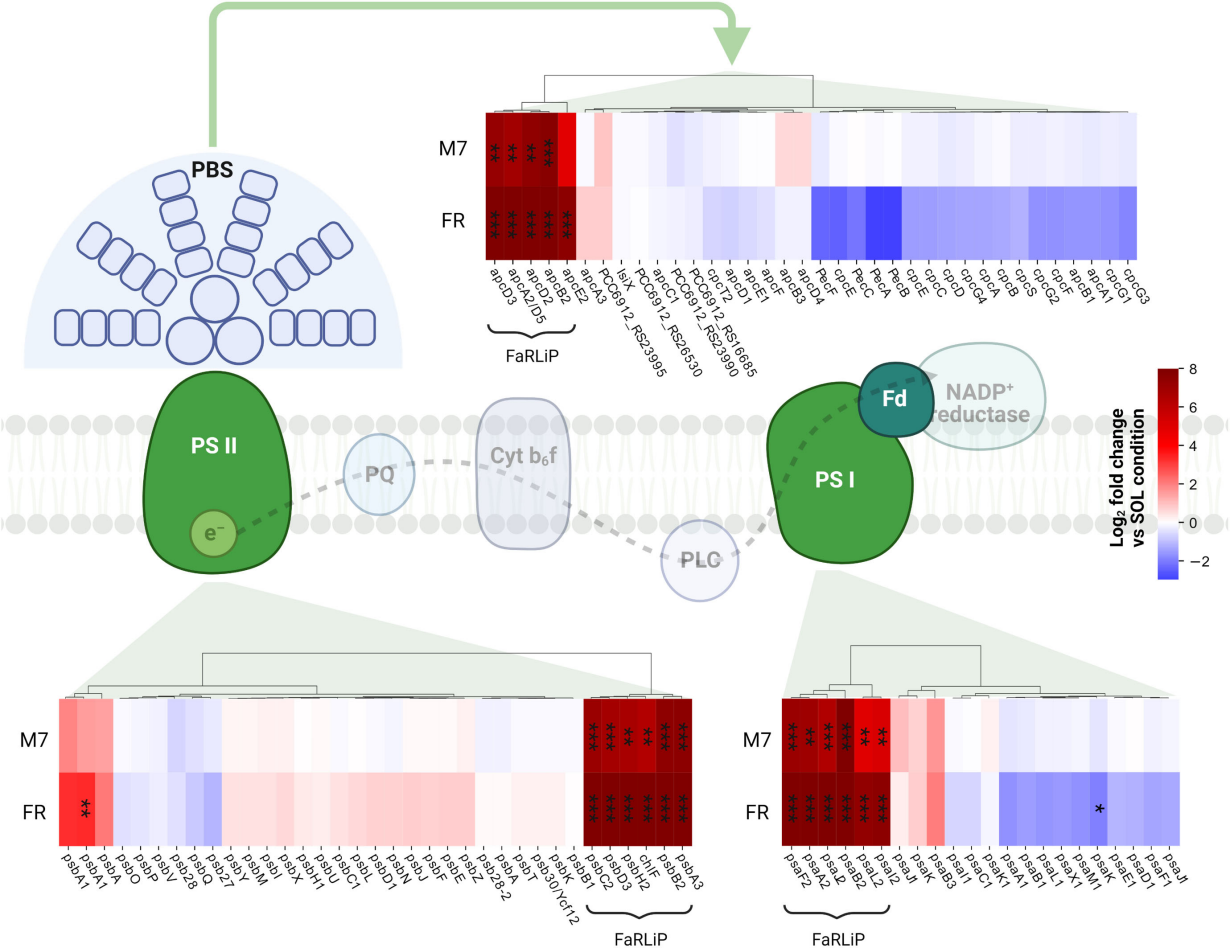
Changes in expression of genes related to photosynthesis. The LFC values of photosystem and phycobilisome genes in M7 and FR compared to SOL are shown. Genes belonging to the FaRLiP group are highlighted. The statistical significance of individual LFCs is highlighted as follows: *, q<0.05; **, q<10^-3^; ***, q<10^-5^. PSII, photosystem II; PSI, photosystem I; PBS, phycobilisome; PQ, plastoquinone; PLC, plastocyanin; Fd, ferredoxin. SOL, Solar light; M7, M-dwarf light; FR, Far-red light. Created with BioRender.com.

In PCC6912, most genes with significant changes (Q< 0.05) were those related to the FaRLiP cluster in M7, indicating that the FaRLiP response is actually triggered early on in this condition, while significant changes in genes from other several pathways were found in FR, showing FaRLiP in this condition is concerted with broader changes in the metabolism. In FR samples compared to SOL, significant upregulation (LFCs ~7-10) was detected in genes from the FR-specific photosystems and phycobilisome (**Figures 4A**, **5**; **Supplementary file S1**). Importantly, the expression of *psbA4*, encoding the chlorophyll *f* synthase ChlF, and the regulatory genes of the FaRLiP cluster *rfpABC*, significantly increased as well. Instead, M7 samples compared to SOL showed a less pronounced upregulation of the FaRLiP cluster (LFCs ~5-7), while the *rfpABC* cluster was not significantly upregulated, although a LFC of 2 was obtained for *rfpB* (**Supplementary file S1**). Overall, these observations are highly consistent with a previous molecular analysis of the closely related cyanobacterial strain *Chlorogloeopsis fritschii* sp. PCC9212 under far-red (Zhao et al., 2015; Ho and Bryant, 2019). In addition, the results presented here show the activation of the FaRLiP response at a molecular level in M7. Importantly, this activation was not detectable from biochemical analyses (**Figure 2**; **Supplementary Figure 3**). In line with these results, previous biochemical and spectroscopic analyses evidenced a slower activation of the FaRLIP response in M7 and the coexistence of far-red and visible-absorbing photosystems (Battistuzzi et al., 2023). The lower activation of the FaRLiP response in M7 may result from the presence of visible light in addition to far-red, or from the different amounts of far-red photons in M7 with respect to FR (20 and 30 µmol of photons m^-2^ s^-1^, respectively). The induction of FaRLiP is indeed based on the ratio between visible and far-red light (Airs et al., 2014; Gan et al., 2014a; Zhao et al., 2015; Ho and Bryant, 2019; Silkina et al., 2019); moreover, the presence of visible light (in particular red light) in addition to far-red light can interfere with the sensitivity of the RfpABC photosensory system (Liu et al., 2023). In the same study, also a dependence of the FaRLiP response to low far-red light intensity has been identified. Finally, under only far-red light, far-red and visible-absorbing photosystems coexist during FaRLiP (Ho et al., 2020). Given this information, in M7 it’s plausible that the lower ratio of red to far-red light causes a lower accumulation of RfpA proteins in the active state, resulting in lower expression of the FaRLiP gene cluster and slower response. Whether the FaRLiP response in M7 is slower or simply less intense remains to be clarified. Interestingly, a *cas6* gene (PCC6912_RS10755) upstream of the FaRLiP cluster was found to have the same expression patterns as FaRLiP genes (**Figure 4A**; **Supplementary Figure 5**, **Supplementary file S1**). However, no upregulation was detected in the rest of the CRISPR-Cas cluster. The strain *C. fritschii* sp. PCC9212, closely related to PCC6912, has an identical CRISPR-Cas cluster close to the FaRLiP cluster, but in the work of Ho and Bryant (Ho and Bryant, 2019), the *cas6* gene was not found to be upregulated. Therefore, the possible role of *cas6* upregulation within FaRLiP response remains unclear and is to be further investigated.

The response of PCC6912 to FR involved other genes related to photosynthesis (**Figure 4A**; **Supplementary file S1**). In particular, *psbA*, encoding the photosystem II subunit D1 was significantly upregulated (LFC ~3, Q< 0.05) in FR compared to SOL. Conversely, ferredoxin *petF* and light-independent protochlorophyllide reductase subunit *chlN* were downregulated in FR (LFCs ~-4 and ~-2, respectively). The *petF* downregulation under FR has been reported before in plants (Yang et al., 2018) and cyanobacteria (Ho and Bryant, 2019) and points towards the decrease of the linear electron transport, since cells cannot efficiently utilize far-red photons yet. Together with *chlN*, also two neighboring genes, part of the same operon, *chlL* (another protochlorophyllide reductase subunit) and PCC6912_RS14015, showed similar changes in their relative transcription levels. The co-regulation of the three genes leads to the hypothesis that PCC6912_RS14015 may also be involved in porphyrin metabolism. Interestingly, *chlB*, encoding the last subunit of the enzyme, was not differentially expressed as *chlN* and *chlL*. The NB-protein (ChlN-ChlB) is reported to be the catalytic component of the complex, while ChlL serves as the electron donor to the NB-protein complex (Muraki et al., 2010). A downregulation of *chlN* was reported by Ho and colleagues (Ho and Bryant, 2019), and could reflect the re-routing of the metabolism towards the synthesis of a small amount of chlorophyll *f* instead of chlorophyll *a*.

Aside from genes related to photosynthesis, an overall upregulation of genes involved in the degradation of fatty acids was observed (**Figure 4A**; **Supplementary file S1**). A similar response was reported in the work of Ho and Bryant (2019), and was proposed to be related to the acquisition of energy and reducing power in FR, when cells are not able to harvest far-red light yet. Interestingly, a canonical *β*-oxidation pathway seems to be absent in cyanobacteria (Figueiredo et al., 2021). Moreover, genes for the biosynthesis *de novo* of fatty acids (*fab genes*) were not differentially expressed in any condition; finally, the gene *fadD* appeared to be significantly upregulated in FR compared to SOL (LFC ~2). KEGG data annotate this gene as encoding a long-chain acyl-CoA synthetase. The ortholog of this gene in PCC6803 is *aaS* (*slr1609*), which was demonstrated to have an important role in the recycling of fatty acids (Kaczmarzyk and Fulda, 2010). Therefore, PCC6912 cells in FR could be recycling fatty acids rather than degrading them to restructure the thylakoid membranes and prepare to host far-red acclimated photosystems. Some genes related to molecule transport were also downregulated, namely *exbB* (PCC6912_RS09125)*, exbD* (PCC6912_RS09130), and *tonB* (PCC6912_RS09120) (**Supplementary file S1**). Interestingly, these genes are part of a single complex related to the transport of a wide range of nutrients (Krewulak and Vogel, 2011). This kind of regulation is expected since, in FR, cells cannot harvest energy and cannot assimilate nutrients. Finally, a cluster of genes with roles in the biosynthesis of lipopolysaccharides (LPS) and the cell wall were downregulated in FR (LFC ~-3 to ~-5; **Supplementary file S1**). The LPS have been observed to have an important role in the membrane integrity of many Gram-negative bacteria, and in some cases, genes for LPS synthesis are essential for cell viability (Saha et al., 2022). In general, bacterial membranes undergo modifications when metabolic or environmental conditions change. This is also coherent with the lack of growth under FR, which implies that no division of the cells is necessary.

When considering atmospheric conditions, significant upregulation was observed in a group of six genes (LFC ~3-6; **Figure 4A**; **Supplementary file S1**). It is worth noting that these genes were upregulated only in FR and ATM MOD. These genes can therefore be considered specific to these two conditions combined. The gene *desC3* is reported to encode a fatty acid desaturase, *inhA* a cyclohexyl-isocyanide hydratase and *hpsU*, an hormogonium polysaccharide biosynthesis acetyltransferase. Hormogonia are reported to be developed by PCC6912 (Hindák, 2008), and are specialized cells which do not divide and grow but retain photosynthetic activity to provide energy for motility (Castenholz et al., 2001). Hormogonia are produced by certain filamentous, heterocyst-forming cyanobacteria to optimize their light environment through phototaxis (Castenholz et al., 2001). The gene *hpsU*, with two other genes (*hpsS* and *hpsT*), is part of a gene cluster which is expressed in developing hormogonia of the cyanobacterium *Nostoc punctiforme* ATCC29133 (Zuniga et al., 2020a). PCC6912_RS21285 and another gene, PCC6912_RS21295, are adjacent to *hpsU*. PCC6912_RS21295 was upregulated (LCF ~4) when comparing ATM TER and ATM MOD in FR, PCC6912_RS21285 was upregulated as well (LCF ~3), even if not significantly. When aligning these sequences with *hpsT* and *hpsS* genes from *N. punctiforme* ATCC29133 (Zuniga et al., 2020a) an identity of 85% was found between PCC6912_RS21285 and *hpsT*, but no similarities were found between PCC6912_RS21295 and *hpsS*. A similarity of 82% was instead found between *hpsS* and PCC6912_RS21280, another adjacent gene not differentially regulated in any condition (**Supplementary file S1**). PCC6912_RS21280 and PCC6912_RS21285 could therefore be respectively *hpsS* and *hpsT*. The upregulation of the cluster could be linked to the necessity of the strain, in combined FR and ATM MOD conditions, to differentiate hormogonia and move away from the unfavorable environment in search of visible light, required for growth, at least until the complete activation of the FaRLiP response. However, a more complex response should be expected for hormogonium differentiation (Campbell et al., 2008; Christman et al., 2011; Gonzalez et al., 2019; Zuniga et al., 2020a; Zuniga et al., 2020b). In previous studies on the cyanobacterium *N. punctiforme* (Gonzalez et al., 2019; Zuniga et al., 2020a; Zuniga et al., 2020b) authors observed an upregulation of the main controllers of hormogonium differentiation, which are the master regulator HrmK, encoded by the *hrmK* gene, and the sigma factors involved in the subsequent hierarchical cascade, encoded by the genes *sigJ*, *sigC*, *sigF*. It was not possible to find a *hrmK* gene ortholog annotated in the PCC6912 genome of this study and a protein blast between *N. punctiforme* sequence and the PCC6912 genome gave inconclusive results; instead, the ortholog genes for *sigJ*, *sigF* and *sigC* in the PCC6912 genome were found. The *sigJ* gene was overexpressed under FR in ATM TER but not significantly, *sigF* and *sigC* genes were instead not overexpressed in any condition tested. The hormogonium differentiation therefore seems not induced, but without information on the *hrmK* gene a definitive proof is lacking. Also, in the *N. punctiforme* study the overexpression of *hrmK, sigJ, sigF* and *sigC* genes were observed at 18 hours and in different experimental conditions (Zuniga et al., 2020b), so it is possible that after 48 hours, in our conditions, the expression of these genes could be different.

### Most differentially regulated genes in PCC6803

In PCC6803, no significant variations for any analyzed gene appeared when comparing M7 to SOL (**Figure 4B**). PCC6803 surprisingly shows the same transcriptional profile for both light spectra. However, significant variations in expression were detected for several clusters of genes when comparing FR to SOL (Q< 0.05) (**Supplementary file S2**). About half of the differentially regulated genes are unknown or uncharacterized. The most differentially regulated genes appeared not to be related to photosynthesis, except for *cpcL*, a paralog of a green-light-induced phycobilisome linker protein encoded by *cpcG*. The proteins encoded by these genes connect the phycobilisome to PSI under green light, redistributing the harvested energy between photosystems (Sanfilippo et al., 2019). The gene *cpcL* was downregulated in FR (LFC ~-4), but not in M7. This result is consistent with previous 77K fluorescence spectroscopy data obtained from FR and M7 long-term acclimated cultures of PCC6803 (Battistuzzi et al., 2023). Under FR, PSI is over-excited due to photons above 700 nm. PCC6803 responds in the long term by changing the relative amounts of PSI and PSII to rebalance the excitation energy of the harvested light. The short-term response highlighted by molecular data, however, is reduction of the relative amounts of CpcL. A lower amount of linker would reduce the redistribution of energy between the two photosystems, conveying the harvested energy mostly to photosystem II, which is less excited under FR. Under M7 however, the presence of visible light in addition to far-red is sufficient to rebalance the excitation energy between the photosystems without the need for CpcL underexpression.

Many of the top differentially regulated genes in FR compared to other lights were involved in nitrogen metabolism (**Figure 4B**; **Supplementary file S2**). The genes *nrtABCD*, *nirA*, and *narB* were all significantly downregulated in FR compared to M7, instead, *norB* was upregulated in that condition. When comparing FR to SOL, among the same genes, the relative transcript levels of *norB* and *nirA* were low (LFC< 2) while those of *narB* and *nrtC*, although higher (LFC ~2), were not significant. Finally, the genes *gifA* and *gifB* were all significantly upregulated in FR when compared to both M7 and SOL (LFC ranging from ~3 to ~4). The operon *nrtABCD* encodes the subunits of a nitrate (NO_3_^-^) transport system protein, which allows the entrance of extracellular NO_3_^-^ inside the cell. The gene *nirA* encodes a ferredoxin-nitrite reductase, while *narB* a ferredoxin-nitrate reductase, and together these proteins form the assimilatory NO_3_^-^ reduction pathway, which allows the conversion of the extracellular NO_3_^-^ to ammonia (NH_4_^+^), which can be used by the cell (Ohashi et al., 2011). Interestingly, the key enzyme of N assimilation, glutamine synthetase (GS, encoded by *glnA* gene), which catalyzes the reaction that converts glutamate to glutamine (Klähn et al., 2018), was not differentially regulated at significant levels in any condition tested. The inhibition of GS indeed takes place at the post-translational level, through the inhibitory factors IF7 and IF17, encoded respectively by the genes *gifA* and *gifB* (Klähn et al., 2018; Perin et al., 2021). The upregulation of *norB* instead is not clear. This gene encodes for a nitric oxide (NO) reductase, even if PCC6803 lacks a functional denitrification pathway able to fix atmospheric N_2_ (Büsch et al., 2002). Yet, *norB* provides a scavenging mechanism for NO, a signal and stress molecule detrimental to the photosynthetic apparatus (Solymosi et al., 2022). An upregulation of *norB* in FR could derive from the necessity to protect photosynthetic proteins from stress. A nitrite reductase (NO-forming) in PCC6803 does not exist (Büsch et al., 2002), so there would be no way to convert NO_2_^-^ to NO and subsequently to nitrous oxide (N_2_O) through nitric oxide reductase. However, it has been demonstrated that PCC6803’s cyanobacterial hemoglobin (*glbN* gene) can serve as an anaerobic nitrite reductase in hypoxic conditions (Sturms et al., 2011), by-passing the lack of a canonical nitrite reductase (NO-forming) and producing toxic NO. Upregulation of *norB* in FR versus M7 was significant in ATM TER while in ATM MOD an LFC of ~-2 was observed, but lacked statistical significance. PCC6803 in FR is always exposed to low O_2_ concentrations: in ATM TER O_2_ consumption exceeds O_2_ production (**Figure 1**), leading to local hypoxia in the cell; in addition, in ATM MOD the atmosphere itself is anoxic. Taken together, these data suggest that, upon exposure to FR, PCC6803 stops the assimilation of NO_3_^-^ (or nitrite, NO_2_^-^) from the environment, since it cannot obtain energy from photosynthesis and utilize it to fix nitrogen into carbon compounds. Nevertheless, the inhibition of the nitrogen assimilatory pathway leads to the accumulation of NO_2_^-^, toxic for the cell and the photosynthetic apparatus, at high concentrations (Zhang et al., 2017; Kocour Kroupová et al., 2018). Therefore PCC6803, through the upregulation of *norB* under FR, could be able to detoxify both NO_2_^-^ and NO.

Other genes differentially regulated included the *hofG* genes, part of a general secretion pathway protein, *wcaL*, *enhA*, *sll1946*, involved in the synthesis of the cell wall and of lipopolysaccharides (LPS) (**Figure 4B**; **Supplementary file S2**) Two copies of *hofG* were significantly downregulated in FR compared to M7 and SOL (LFC ranging from ~2 to ~3), the third instead showed significant downregulation only when comparing FR to SOL in ATM TER (LFC ~2). The gene *wcaL* was significantly downregulated in FR with respect to M7 and SOL (LFC from ~2 to ~3), *enhA* and *sll1946* instead were significantly upregulated in the same conditions (LFC ~3, ~2 in ATM MOD). These are once again plausible responses related to the stop of growth under FR, due to the insufficient light for photosynthesis.

Finally, when comparing atmospheric compositions, albeit differentially regulated genes were found, their LFC rarely exceeded the threshold values (LFC > 2 or< -2) and were never significant (**Figure 4B**; **Supplementary file S2**). These results differ from previous reports, where several gene clusters were found to be upregulated under low oxygen conditions (Summerfield et al., 2008; Summerfield et al., 2011). The discrepancy could be explained by the different conditions employed for the experiments. In our experiments, in all light conditions, the initial anoxic atmosphere evolved, after 48 h, to an atmosphere containing some amounts of O_2_, due to oxygenic photosynthesis (**Figure 1**). The added O_2_, even in FR, could be sufficient to avoid the upregulation of low-oxygen-related genes. Conversely, in their work (Summerfield et al., 2008), the culture was continuously bubbled with 99.9% N_2_ and 0.1% CO_2_, flushing away the O_2_ evolved by cyanobacteria and maintaining very low-O_2_ conditions. Also, the authors investigated the response of the strain to oxygen depletion after 1, 2 and 6 hours, a very short time if compared to our study. This could explain why most of the low-O_2_-induced genes were not found differentially regulated in this study.

## Conclusions

This study presents novel molecular insights into the acclimation strategies of two cyanobacteria, *C. fritschii* PCC6912 and *Synechocystis* sp. PCC6803, under the combined conditions of simulated light spectra and atmospheres. Surprisingly, an initial anoxic condition does not play a great role in the acclimation of both strains, as a very low number of differentially regulated genes was found when comparing atmospheres. Light instead plays a major role in determining the transcriptional responses of the strains. On one hand, when comparing solar and M-dwarf simulated lights, only genes involved in the FaRLiP response show a different transcriptional profile in PCC6912. The additional far-red photons in the spectrum are sensed and an acclimation response is pursued, enhancing the harvesting capabilities of PCC6912. Under only far-red, apart from the FaRLiP cluster, other pathways gain importance, in addition to what was observed previously in *C. fritschii* PCC 9212 (Ho and Bryant, 2019). These pathways are involved together in the reduction of the cell metabolism and division under far-red as long as the FaRLiP acclimation is not complete and it is not possible to harvest photons for photosynthesis. When combining far-red light and an anoxic atmosphere though, in PCC6912 an escape strategy from adverse environmental conditions is observed. On the other hand, the transcriptional profile of PCC6803 under both simulated stellar lights is identical, the strain does not perform relevant acclimation responses. Under far-red light, however, due to the scarcity of visible light, a great transcriptional response is observed, once again concerning a general reduction in the cell metabolism to reduce energy consumption. Finally, in both strains under far-red light, many differentially regulated genes are not yet characterized, limiting our capability to comprehensively describe the metabolic response to this light spectrum. These molecular data confirm previous physiological results (Claudi et al., 2021; Battistuzzi et al., 2023): it was shown that specific acclimations to far-red are not necessary to harvest a simulated M-dwarf light spectrum. However, if far-red photons are available and the cyanobacterium is capable of utilizing them, an acclimation response towards that goal is initiated. Furthermore, the absence of oxygen minimally impacts in the short-term the transcriptional response of the strains, showing that cyanobacteria are ancestrally equipped to cope with anoxia. Taken together these results prove the adaptability and versatility of cyanobacteria and are a positive signal for the search for oxygenic photosynthesis on exoplanets orbiting M-dwarf stars.

## Data availability statement

The datasets presented in this study can be found in online repositories. The names of the repository/repositories and accession number(s) can be found below:BioProject: PRJNA1028124.

## Author contributions

MB: Writing – original draft, Data curation, Formal analysis, Investigation, Methodology, Resources, Validation, Visualization, Writing – review & editing. MM: Data curation, Formal analysis, Methodology, Resources, Software, Validation, Visualization, Writing – original draft, Writing – review & editing. LC: Data curation, Formal analysis, Methodology, Resources, Software, Validation, Visualization, Writing – review & editing. LiT: Conceptualization, Methodology, Validation, Writing – review & editing. LaT: Conceptualization, Methodology, Resources, Software, Supervision, Validation, Writing – review & editing. SC: Conceptualization, Methodology, Resources, Software, Supervision, Validation, Writing – review & editing. RC: Methodology, Resources, Software, Validation, Writing – review & editing. LP: Funding acquisition, Methodology, Resources, Validation, Writing – review & editing. NL: Conceptualization, Funding acquisition, Methodology, Project administration, Resources, Supervision, Validation, Writing – review & editing.
