# Supplementary material for: Transcriptomic and photosynthetic analyses of Synechocystis sp. PCC6803 and Chlorogloeopsis fritschii sp. PCC6912 exposed to an M-dwarf spectrum under an anoxic atmosphere
Source: Front Plant Sci. 2024 Jan 18;14:1322052. doi: 10.3389/fpls.2023.1322052 (PMC10830646; doi:10.3389/fpls.2023.1322052)
Supplement: Supplementary file 1 [file DataSheet_1.pdf]

Supplementary Material

Transcriptomic and photosynthetic analyses of *Synechocystis* sp. PCC6803 and *Chlorogloeopsis fritschii* sp. PCC6912 exposed to an M-dwarf spectrum under an anoxic atmosphere

M. Battistuzzi[1,2,3,*], M. S. Morlino[2], L. Cocola[1], L. Trainotti[2], L. Treu[2], S. Campanaro[2], R. Claudi[4,5], L. Poletto[1] and N. La Rocca[2,3]

[1] National Council of Research of Italy, Institute for Photonics and Nanotechnologies (CNR-IFN), Padua, Italy,
[2] Department of Biology, University of Padua, Padua, Italy,
[3] Center for Space Studies and Activities (CISAS), University of Padua, Padua, Italy,
[4] National Institute for Astrophysics, Astronomical Observatory of Padua (INAF-OAPD), Padua, Italy
[5] Department of Mathematics and Physics, University Roma Tre, Rome, Italy.

*** Correspondence:**
Mariano Battistuzzi

mariano.battistuzzi@unipd.it

Keywords: Cyanobacteria, Laboratory simulation experiments, M-dwarf spectrum, Anoxia, Transcriptomic Analysis, RNA-seq, FaRLiP.

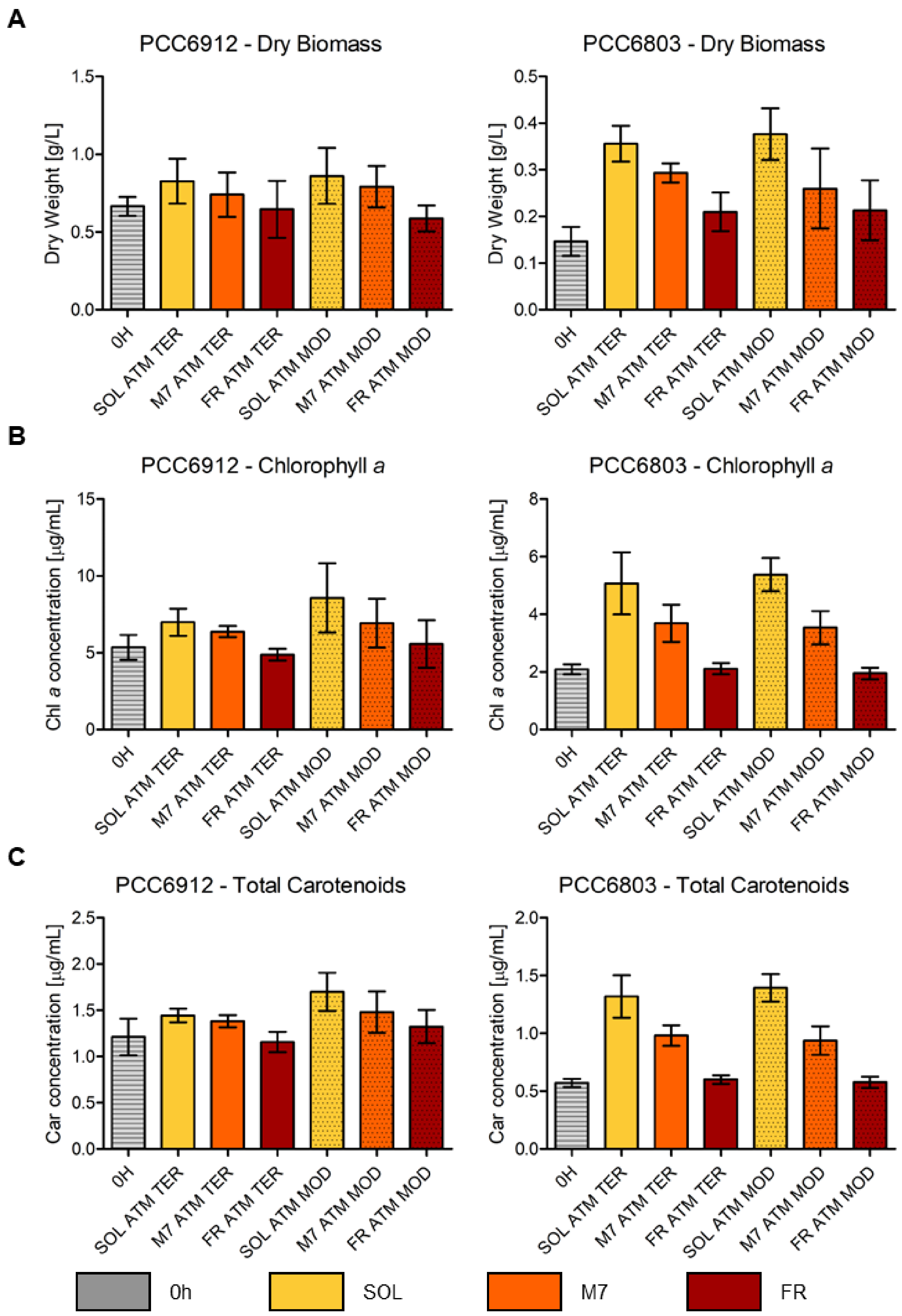

Figure S1. Dry biomass (A), Chlorophyll *a* (B) and total carotenoids (C) content of PCC6912 and PCC6803 after 48 h of exposure to the different environmental conditions. A statistical analysis of the results is provided in Table S2. SOL, Solar light; M7, M-dwarf light; FR, Far-red light; ATM TER, oxic atmosphere; ATM MOD, anoxic atmosphere.

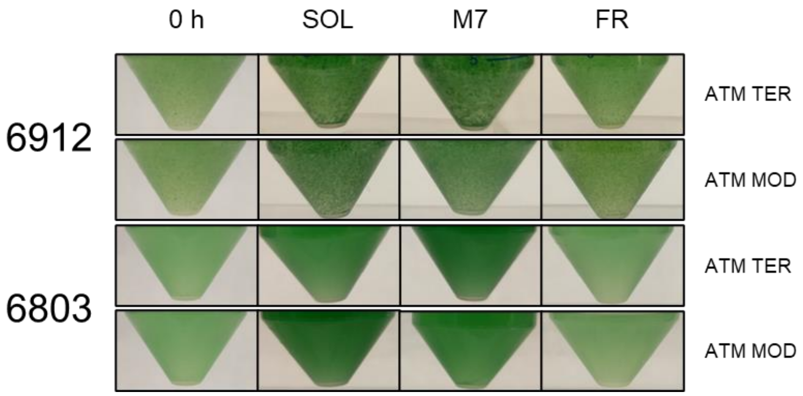

Figure S2. cultures of PCC6803 and PCC6912 at 0 and 48 h in the tested conditions. SOL, Solar light; M7, M-dwarf light; FR, Far-red light; ATM TER, oxic atmosphere; ATM MOD, anoxic atmosphere.

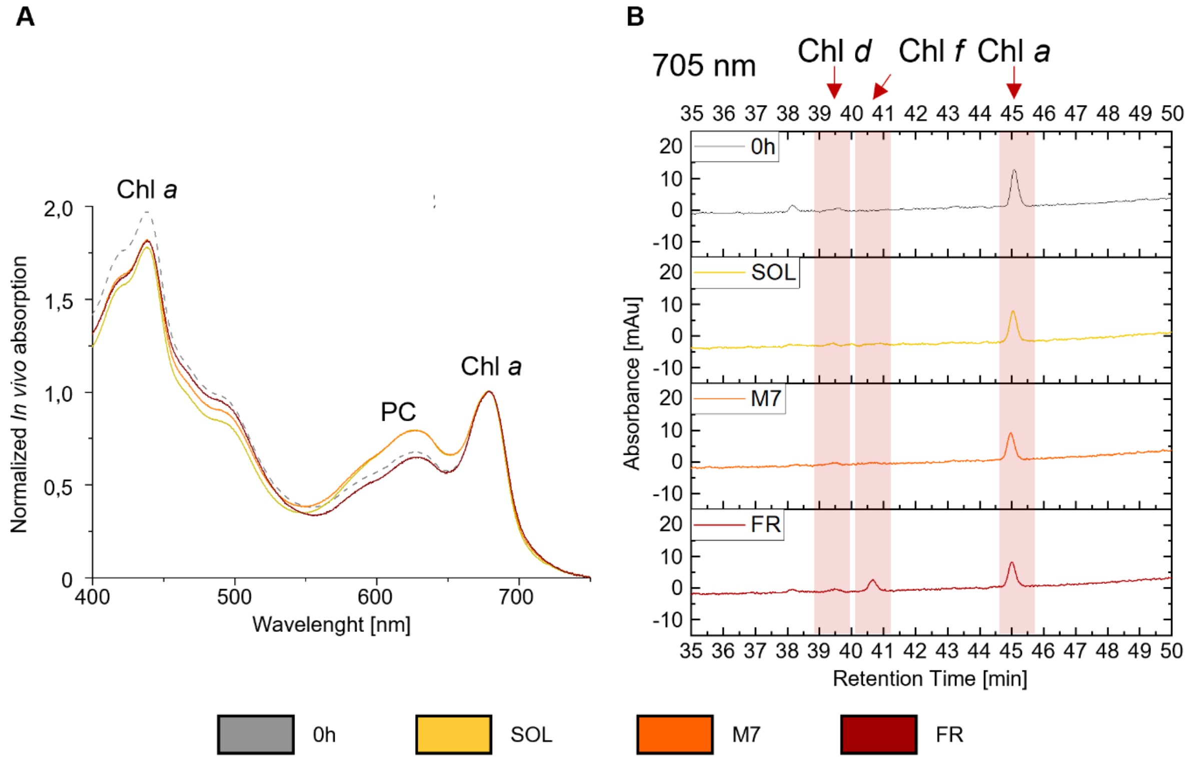

Figure S3. A) example of *In vivo* absorption spectra of PCC6912 grown for 48 h in the three light conditions tested under ATM MOD. Spectra are normalized at 680 nm; B) example HPLC chromatograms at 705 nm of PCC6912 after 48 h of exposure to the different light conditions under ATM MOD. Retention times for chlorophylls *a*, *d*, and *f* are highlighted with a red band. SOL, Solar light; M7, M-dwarf light; FR, Far-red light; ATM MOD: anoxic atmosphere.

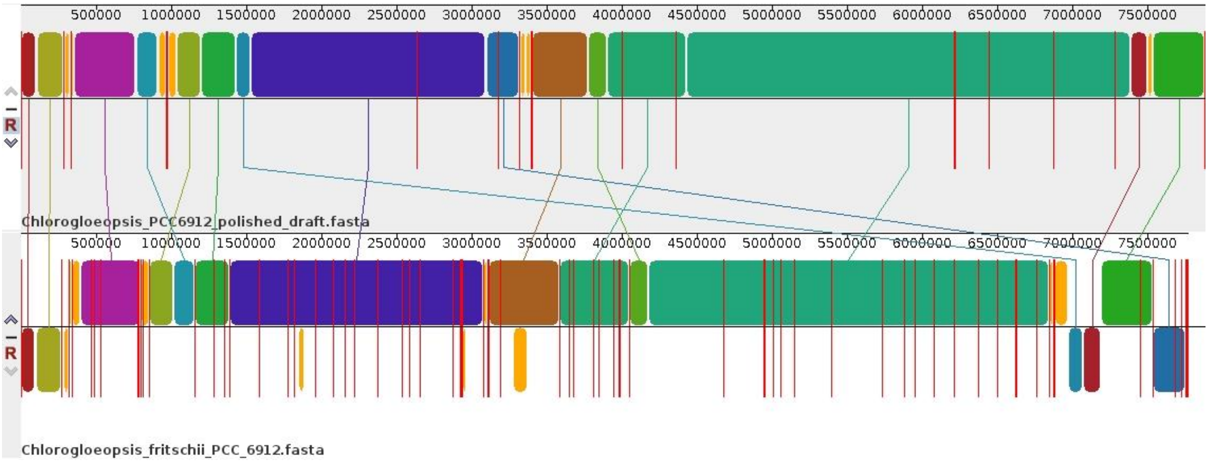

Figure S4. Mauve alignment of the PCC6912 *de novo* assembly (top) with the reference genome GCA_003990575.1 (bottom). Red lines delimit contigs, while homologous regions are displayed with corresponding color blocks and linked by a line. Regions with the same orientation as the *de novo* assembled genome are visualized on top of the GCA_003990575.1 track, while inverted regions are displayed at the bottom.

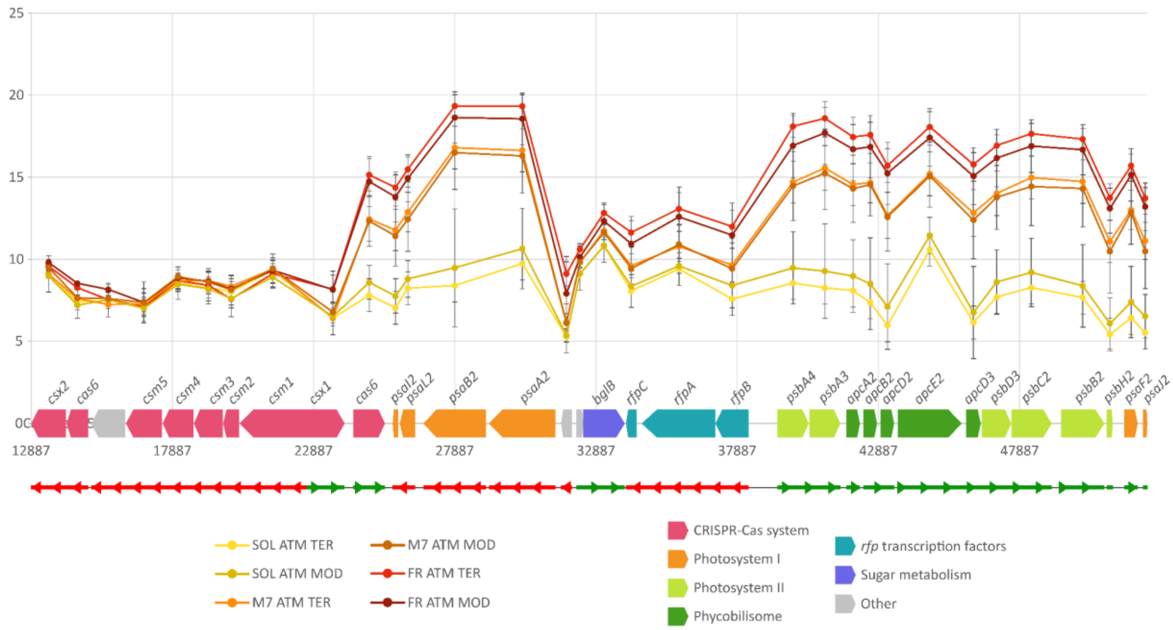

Figure S5. Gene structure of the locus coding for one CRISPR/Cas system and the neighbouring FaRLiP gene cluster. For each gene, the average VST-normalised expression in the six conditions is shown in the Y axis. Operon structure is displayed at the bottom, with sense operons in green and antisense operons in red.

Table S1. Experimental design of the study. For each experiment, 3 biological replicates were obtained.

Experiment	Organism	Light Spectrum	Atmosphere Composition
1	PCC6803	M7	ATM TER
2	PCC6803	SOL	ATM TER
3	PCC6803	FR	ATM TER
4	PCC6803	M7	ATM MOD
5	PCC6803	SOL	ATM MOD
6	PCC6803	FR	ATM MOD
7	PCC6912	M7	ATM TER
8	PCC6912	SOL	ATM TER
9	PCC6912	FR	ATM TER
10	PCC6912	M7	ATM MOD
11	PCC6912	SOL	ATM MOD
12	PCC6912	FR	ATM MOD

Table S2. statistical analyses for Figure S1. (one-way ANOVA, p-value< 0,05). Significant p-values are highlighted.

	PCC6912			PCC6803		
	DW	Chl *a*	Tot Car	DW	Chl *a*	Tot Car
Tukey's multiple comparisons test	Adj. P Value	Adj. P Value	Adj. P Value	Adj. P Value	Adj. P Value	Adj. P Value
0H vs. SOL ATM TER	0,1965	0,1175	0,1136	<0,0001	<0,0001	<0,0001
0H vs. M7 ATM TER	0,9118	0,6191	0,4201	<0,0001	<0,0001	<0,0001
0H vs. FR ATM TER	>0,9999	0,9842	0,9947	0,1558	>0,9999	0,9962
0H vs. SOL ATM MOD	0,0301	<0,0001	<0,0001	<0,0001	<0,0001	<0,0001
0H vs. M7 ATM MOD	0,2854	0,0825	0,0185	0,0008	<0,0001	<0,0001
0H vs. FR ATM MOD	0,8523	0,9997	0,8442	0,3634	0,9981	>0,9999
SOL ATM TER vs. M7 ATM TER	0,9116	0,9768	0,9963	0,361	0,0012	<0,0001
SOL ATM TER vs. FR ATM TER	0,2322	0,0663	0,0861	0,0003	<0,0001	<0,0001
SOL ATM TER vs. SOL ATM MOD	0,9989	0,3076	0,1653	0,9918	0,9563	0,8313
M7 ATM TER vs. FR ATM TER	0,8789	0,3646	0,2905	0,0717	0,0002	<0,0001
M7 ATM TER vs. M7 ATM MOD	0,9871	0,9813	0,9449	0,8956	0,9989	0,9868
FR ATM TER vs. FR ATM MOD	0,9801	0,9528	0,6492	>0,9999	0,9984	0,9996
SOL ATM MOD vs. M7 ATM MOD	0,9217	0,197	0,2434	0,0033	<0,0001	<0,0001
SOL ATM MOD vs. FR ATM MOD	0,0023	0,0023	0,0083	0,0006	<0,0001	<0,0001
M7 ATM MOD vs. FR ATM MOD	0,0294	0,4193	0,6421	0,8218	0,0002	<0,0001

Table S3. Genome statistics of the assembly of PCC6912 used in this study, accession number JAWJEG000000000. The main metrics to assess genome quality are reported, along with completeness and contamination assessed via CheckM2, and number of detected genes.

**Number of contigs**	23
**Length**	7.89 Mb
**N50**	640 kbp
**GC%**	41.53
**Median depth of coverage**	123
**CheckM2 completeness**	100
**CheckM2 contamination**	0.74
**Total genes**	6823
**Liftoff genes from GCA_003990575.1**	6760
**De novo genes**	63

Table S4. Calculation of the Average Nucleotide Identity (ANI) with dREP.

Reference	Reference length	Reference #contigs	Reference N50	Query	Query length	Query #contigs	Query N50	Alignment length	Reference coverage	ANI
**GCA_003990575.1**	7.77 Mbp	85	165 kbp	**PCC6912 de novo**	7.89 Mbp	23	640 kbp	7.782.379	1.002	99.97 %
